# Supplementary material for: The interactome of intact mitochondria by cross-linking mass spectrometry provides evidence for coexisting respiratory supercomplexes
Source: Mol Cell Proteomics. 2017 Dec 8;17(2):216–32. doi: 10.1074/mcp.RA117.000470 (PMC5795388; doi:10.1074/mcp.RA117.000470)
Supplement: Supplemental Data [file supp_RA117.000470_133922_0_supp_23978_fzffwf.zip › spectra_annotation/mito_DR_spectra_annotation/265-1-10-1-8-1.pdf]

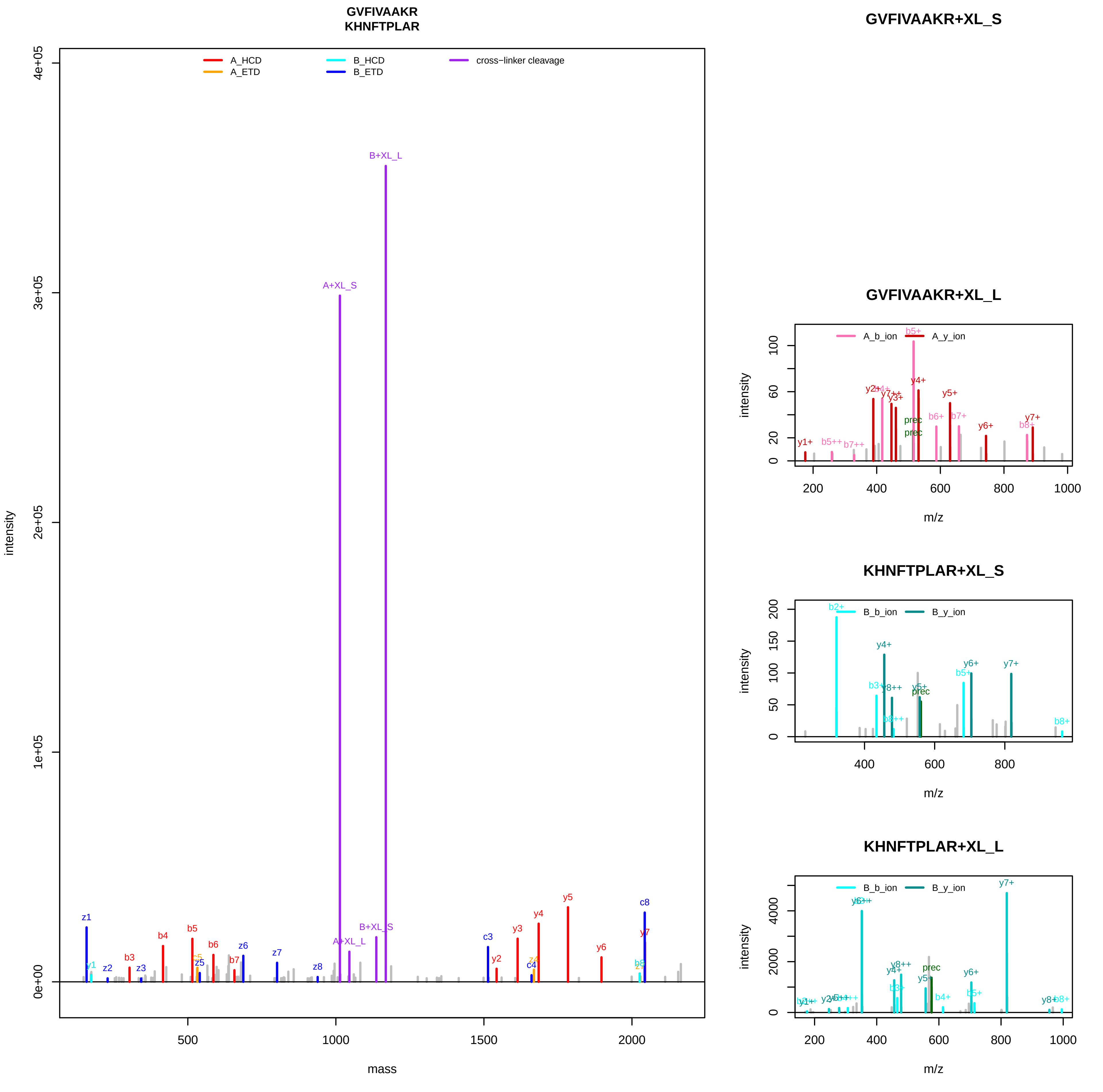
GVFIVAAKR
KHNFTPLAR
A_HCD
A_ETD
B_HCD
B_ETD
cross−linker cleavage
B+XL_L
A+XL_S
B+XL_S
A+XL_L
z1
y1
z2
b3
z3
b4
b5
z5
b6
b7
z6
z7
z8
c3
y2
y3
c4
y4
y5
y6
c8
y7
intensity
mass
0e+00
1e+05
2e+05
3e+05
4e+05
500
1000
1500
2000
GVFIVAAKR+XL_S
GVFIVAAKR+XL_L
A_b_ion
A_y_ion
b5+
y1+
b5++
b7++
y2+
y7++
y3+
y4+
prec
b6+
b7+
y5+
y6+
b8+
y7+
intensity
m/z
KHNFTPLAR+XL_S
B_b_ion
B_y_ion
b2+
y4+
b3+
y8++
b8++
y5+
prec
b5+
y6+
y7+
b8+
KHNFTPLAR+XL_L
y7+
y6+
y8++
y4+
y5+
b3+
b4+
b5+
y8+
b8+
y1+
y2+
prec
y6+
0
20
60
100
50
150
200
2000
4000
200
400
600
800
1000
